# Quality Evaluation of Human Bone Marrow Mesenchymal Stem Cells for Cartilage Repair

**DOI:** 10.1155/2017/8740294

**Published:** 2017-08-01

**Authors:** Katsunori Shiraishi, Naosuke Kamei, Shunsuke Takeuchi, Shinobu Yanada, Hisashi Mera, Shigeyuki Wakitani, Nobuo Adachi, Mitsuo Ochi

**Affiliations:** ^1^Department of Orthopaedic Surgery, Division of Medicine, Biomedical Sciences Major, Graduate School of Biomedical Sciences, Hiroshima University, Hiroshima, Japan; ^2^Medical Center for Translational & Clinical Research, Hiroshima University Hospital, Hiroshima, Japan; ^3^Japan Tissue Engineering Co., Ltd., Gamagori, Japan; ^4^Uonuma Institute of Community Medicine, Niigata University Medical and Dental Hospital, Niigata, Japan; ^5^Department of Health and Sports Sciences, Mukogawa Women's University, Nishinomiya, Japan

## Abstract

Quality evaluation of mesenchymal stem cells (MSCs) based on efficacy would be helpful for their clinical application. In this study, we aimed to find the factors of human bone marrow MSCs relating to cartilage repair. The expression profiles of humoral factors, messenger RNAs (mRNAs), and microRNAs (miRNAs) were analyzed in human bone marrow MSCs from five different donors. We investigated the correlations of these expression profiles with the capacity of the MSCs for proliferation, chondrogenic differentiation, and cartilage repair in vivo. The mRNA expression of *MYBL1* was positively correlated with proliferation and cartilage differentiation. By contrast, the mRNA expression of *RCAN2* and the protein expression of TIMP-1 and VEGF were negatively correlated with proliferation and cartilage differentiation. However, MSCs from all five donors had the capacity to promote cartilage repair in vivo regardless of their capacity for proliferation and cartilage differentiation. The mRNA expression of *HLA-DRB1* was positively correlated with cartilage repair in vivo. Meanwhile, the mRNA expression of *TMEM155* and expression of miR-486-3p, miR-148b, miR-93, and miR-320B were negatively correlated with cartilage repair. The expression analysis of these factors might help to predict the ability of bone marrow MSCs to promote cartilage repair.

## 1. Introduction

Mesenchymal stem cells (MSCs) have the capacity for self-renewal [1] and differentiation into several mesoderm-type lineages, including osteoblasts, chondrocytes, adipocytes, myocytes, and vascular cells [2] and are considered to be nonimmunogenic [3, 4]. Therefore, MSCs are one of the most promising cellular sources of stem cells that can be studied without using any immunosuppressive drugs, for both research and clinical purposes. Clinical studies using MSCs are widely available. For example, MSCs have been used in the therapy of diseases such as extended cartilage [5, 6] and osseous defects [7], intervertebral disc [8], acute myocardial infarction [9], leukemia [10], and diabetes [11]. We have started two clinical trials of intra-articular injection of autologous bone marrow MSCs for articular cartilage repair based on our previous animal experiments [5, 12, 13]. However, the functional quality of MSCs for cartilage regeneration might be diversified depending on the donor due to the heterogeneity of MSCs. There have been reports that differentiation and proliferation capacity decrease with age [14, 15] and, consequently, the use of autologous MSCs for tissue repair, which in some indications concerns elderly patients, has certain limits [16]. Quality evaluation confirming the properties of MSCs has been established and is based on cell surface markers (negative for CD14 or CD11b, CD19, CD34, CD45, CD79*α*, and HLA-DR and positive for CD73, CD90, CD105, CD166, and CD44 [17–19]) and differentiation capacity [20–22]. However, quality evaluation based on the efficacy of MSCs has been not established. The evaluation criteria for quality of MSCs based on the efficacy would be required for the practical application of MSC transplantation. In this study, we aimed to devise a method for functional quality assessment of MSCs for cartilage regeneration by examining the relationships among the following data in human bone marrow MSCs (hMSCs): profiles of cartilage anabolic and catabolic factors, messenger RNAs (mRNAs), and microRNAs (miRNAs), and the capacity for cell proliferation, chondrogenic differentiation *in vivo*, and cartilage regeneration *in vivo*.

## 2. Materials and Methods

All procedures were approved and performed by the Guide for Animal Experimentation, Hiroshima University, and the Committee of Research Facilities for Laboratory Animal Sciences, Graduate School of Biomedical Sciences, Hiroshima University.

In this study, hMSCs were purchased from Lonza Walkersville Inc. (PT-2501, Walkersville, MD, USA). All these hMSCs passed the quality inspection conducted by Lonza company using cell viability (more than 75%), adipogenic and osteogenic differentiation (Oil Red O staining and calcium deposition staining), and FACS analysis of cell surface markers (more than 90% were positive for CD29, CD44, CD105, and CD166 and negative for CD14, CD34, and CD45). Assays of cell growth rate (GR) and colony formation (CF), pellet cultures *in vitro*, and transplantation of hMSCs into cartilage defect models *in vivo* were performed using hMSCs from five different donors. The age, race, and sex of the five different donors were as follows: 22-year-old black man, 20-year-old white man, 39-year-old black man, 29-year-old white woman, and 41-year-old white woman.

### 2.1. Cultures of hMSCs and Human Skin Fibroblasts

The hMSCs at passage two were centrifuged at 500*g* for 5 min. The cells were resuspended in culture medium composed of Dulbecco's modified Eagle medium (DMEM; Life Technologies, Carlsbad, CA, USA), 15% fetal bovine serum (FBS; SAFC Biosciences Inc., Irvine, CA, USA), 20 mmol/ml of 4-(2-hydroxyethyl)-1-piperazineethanesulfonic acid (Life Technologies), 50 *μ*g/ml gentamycin (Gentacin®, MSD, Tokyo, Japan), and 0.25 *μ*g/ml amphotericin (Fungizon®, Bristol-Myers Squibb, New York, NY, USA). The suspension was then plated into culture dishes. The dishes were incubated in an atmosphere of 95% relative humidity and 5% CO_2_ at 37°C until 70–80% confluency, and the cells were then harvested with Trypsin (TrypLE™ Express; Life Technologies, Carlsbad, CA, USA). After adding high-glucose DMEM® with 10% FBS and 1% antibiotics, the cells were neutralized and harvested by centrifugation at 200*g* for 5 min and the resulting supernatant frozen at −80°C with Cellbanker® 1 (LSI Medience, Tokyo, Japan) until further testing. The cells were defined as passage three (P3). The hMSCs at P3 were reseeded under high-glucose DMEM with 10% FBS and 1% antibiotics to grow the hMSCs. These adherent cells have been referred to as hMSCs at passage four (P4). The hMSCs at P4 were used in the current study. Adult normal human dermal fibroblasts (hFBs; Lonza Japan, Tokyo, Japan) at P2 were cultured with Fibroblast Cell Growth Medium-2 BulletKit (FGM™-2 BulletKit™; Lonza Japan, Tokyo, Japan) until P4 by a similar method and the cells at P4 were used.

### 2.2. Assay of Growth Rate for hMSCs

The hMSCs at P4 were seeded onto culture dishes at 5.0 × 10^3^ cells/cm^2^ in Mesenchymal Stem Cell Basal Medium (MSCBM™; Lonza Japan, Tokyo, Japan) and incubated in an atmosphere of 95% relative humidity and 5% CO_2_ at 37°C. Cell division rate was assessed after 5 days.

### 2.3. Colony-Forming Assay

In a pilot study, we confirmed a positive correlation (*P* < 0.001, *R* = 0.992) between hMSC seeding density and capacity for CF. Because the CF measurement did not reflect the seeding density, the capacity for CF did not have an effect of the secretor factor and could measure quality of cells by CF measurement. The hMSCs at P4 were plated at 1.5 × 10^3^ cells/T75 flask in MSCBM (Lonza Japan) for 14 days. The medium was changed at three- to four-day intervals. After embedding the plate in paraffin, the cells were stained by 1.0% crystal violet solution (Wako, Osaka, Japan) for 10 min. Aggregates of cells differentiated than 50 cells were counted as one colony, and we calculated the ratio of these colonies among all seeded cells.

### 2.4. Analysis of Protein Derived from Culture Supernatant

MSCs at 70–80% confluency were refed with culture media. After 48 h incubation, the media were collected. To remove debris, the media were centrifuged at 600*g* for 5 min and the supernatants were collected as MSC-conditioned media (MSC-CMs). They were stored at −80°C until they were used for the following assays.

The MSC-CMs were analyzed by enzyme-linked immunosorbent assay (ELISA). Sandwich ELISA kits purchased from R&D Systems (Minneapolis, MN, USA) were used for bone morphogenetic protein- (BMP-) 2, BMP-7, fibroblast growth factor- (FGF-) 2, insulin-like growth factor- (IGF-) 1, transforming growth factor- (TGF-) *α*, TGF-*β*1, TGF-*β*2, tissue inhibitor of metalloproteinase- (TIMP-) 1, TIMP-2, platelet-derived growth factor- (PDGF-) AA, interleukin- (IL-) 2, IL-17, monocyte chemotactic protein- (MCP-) 1, matrix metalloproteinase- (MMP-) 1, MMP-3, MMP-9, MMP-13, RANTES, stromal cell-derived factor- (SDF-) 1*α*, macrophage inflammatory protein- (MIP-) 1*α*, MIP-1*β*, and MIP-3*α*. Kits from Life Technologies (Carlsbad, CA, USA) were used for hepatocyte growth factor (HGF), IL-1*β*, IL-4, IL-8, IL-10, TNF-*α*, interferon- (IFN-) *γ*, and vascular endothelial growth factor (VEGF). Assays were performed according to the manufacturer's instructions in duplicate. As a control, culture media were also analyzed. The hFBs at P4 were cultured at the same time and were compared with hMSCs as a control.

### 2.5. The Assessment of Chondrogenic Differentiation Using Pellet Culture for hMSCs

The capacity for chondrogenic differentiation of the hMSCs from each donor was evaluated using pellet culture.

A pellet culture system for chondrogenesis was used [23–26]. About 2.5 × 10^5^ hMSCs at P4 were placed in a 15 ml polyethyleneterephthalate tube (Sumitomo Bakelite) and centrifuged at 450*g* for 10 min. The pellet was cultured at 37°C with 5% CO_2_ in 500 *μ*l of chondrogenic medium containing 500 ng/ml BMP-6 (27) (R&D Systems, Minneapolis, MN, USA) in addition to high-glucose DMEM supplemented with 10 ng/ml TGF-*β*3 (R&D Systems, Minneapolis, MN, USA), 10^−7^ M dexamethasone, 50 *μ*g/ml ascorbate-2-phosphate, 40 *μ*g/ml proline, 100 *μ*g/ml pyruvate (Sigma-Aldrich, St. Louis, MO, USA), and 50 mg/ml ITS+ Premix (Becton Dickinson; 6.25 *μ*g/ml insulin, 6.25 *μ*g/ml transferrin, 6.25 ng/ml selenous acid, 1.25 mg/ml BSA, and 5.35 mg/ml linoleic acid). The medium was replaced every 3 to 4 days for 21 days. For microscopy, the pellets were embedded in paraffin, cut into 5 *μ*m sections, and stained with 0.05% toluidine blue solution (Muto Pure Chemicals Co., Ltd., Tokyo, Japan). The production of extracellular matrix was evaluated by measuring the percentage of metachromasy in pellets (PMP) derived from hMSCs stained with toluidine blue by image processing software (WinROOF®, Mitani, Tokyo, Japan). We calculated the PMP for all areas in pellets.

### 2.6. Real-Time Quantitative Polymerase Chain Reaction (qPCR) of Pellets

Total RNA was isolated from pellets using a Qiagen RNeasy Micro Kit (Qiagen, Valencia, CA, USA). cDNA was synthesized from RNA using Super Script VILO Master Mix (Life Technologies). As a control, total RNA was obtained from normal knee cartilage dissected from skeletally matured cadaveric donors (Articular Engineering, Northbrook, IL, USA). qPCR was performed using Power SYBR Green Master Mix (Life Technologies). cDNA samples were analyzed for both collagen type II (*COL II*) and the reference gene (glyceraldehyde-3-phosphate dehydrogenase (*GAPDH*)). Assays were performed according to the manufacturer's instructions. The primer sequences used for the experiment are shown in Table 1. The amount of *COL II* mRNA expressed was normalized to *GAPDH* expression. In addition, the amount of mRNA expressed in hMSCs from donors (hMSC-1 to hMSC-5) was normalized to expression in hMSC-1 for the purpose of comparing the mRNA expressions among five donors.

### 2.7. The Assessment of Capacity for Cartilage Regeneration Using Cartilage Repair

In this study, male nude rats aged 9 to 10 weeks were used and were anesthetized with an intraperitoneal administration of 1.0 ml/kg sodium pentobarbital before surgery. The medial parapatellar approach was used to expose the knee joint. We created full thickness articular cartilage defects of 2 mm in diameter and 1 mm in depth at the patellar groove of the distal femur using a power drill, and the joint capsule and skin incision were closed with 6-0 nylon sutures. Rats were divided into two groups. In the control group, phosphate-buffered saline (PBS, 25 *μ*l) was injected into the five operated joints (*n* = 5). This group indicated the natural course of healing of the osteochondral defect. In the hMSC group, 3 × 10^5^ hMSCs from the five donors were injected into each operated joint (*n* = 5/donor). After transplantation, all nude rats were allowed to move freely in their cages.

### 2.8. Histological Evaluation

All nude rats were sacrificed by intraperitoneal injection of a lethal dose of pentobarbital sodium at 12 weeks after the injection. The patellar groove was resected and fixed in 4% paraformaldehyde for 24 h. The specimens were then decalcified with 0.5 M ethylenediaminetetraacetic acid. After that, the specimens embedded in paraffin block were cut into 5 *μ*m sections serially along the sagittal plane that included the center of the defect. For histological assessment, these sections were stained with Safranin-O/Fast green. The specimens were graded semiquantitatively. The grading scale was based on a histological grading scale for cartilage regeneration as previously described [27].

### 2.9. 3D-Gene® Human Oligo Chip 25k for mRNA and TaqMan® Low-Density Array for miRNA Expression Profiling

The hMSCs and hFBs at P4 were homogenized on plate using TRIzol Reagent (Life Technologies), and total RNAs were isolated according to the manufacturer's instructions. For mRNA microarray analysis, 3D-Gene Human Oligo chip 25k (3D-Gene; Toray Industries, Tokyo, Japan) was used (24,460 distinct genes). The gene expression of hFBs at P4 was used as a control for normalization. Experimental procedures for TaqMan low-density array analysis (TLDA®; Life Technologies) were performed using TaqMan Array Human microRNA Cards® (card A v2.0 and card B v3.0) according to the manufacturer's instructions to identify differentially expressed miRNAs in hMSCs of each donor.

### 2.10. Statistical Analysis

All assays were performed in triplicate. The results are shown as mean values and standard deviations. Pearson's correlation coefficient calculated using software of the statistic program for Windows by Statcel4® of Excel Statistics (Statcel4: Nebula Company, Bunkyo-ku, Tokyo, Japan) was used to evaluate associations among capacity for cell proliferation, chondrogenic differentiation, and cartilage regeneration, and expression pattern of proteins, mRNAs, and miRNAs in hMSCs. Multiple comparison was performed for the evaluation of histological scores of specimens from the knee of nude rats between each group using Bartlett's test and one-way analysis of variance. When a significant *p* value was found, the Tukey-Kramer method was used to identify significant differences among the groups. The significance level was defined at *P* < 0.05 for all tests.

## 3. Results

### 3.1. Growth Rate and Colony Forming

**Figure 1:**
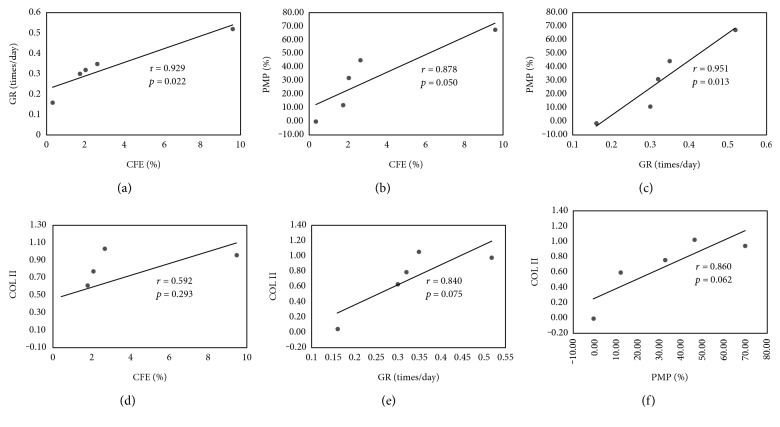
Correlations between (a) CFE and GR, (b) CFE and PMP, (c) GR and PMP, (d) CFE and COLII, (e) GR and COLII, and (f) PMP and COLII were *r* = 0.929 and *P* = 0.022, *r* = 0.878 and *P* = 0.050, *r* = 0.951 and *P* = 0.013, *r* = 0.592 and *P* = 0.293, *r* = 0.840 and *P* = 0.075, and *r* = 0.860 and *P* = 0.062, respectively.

The hMSCs from five different donors were ranked from hMSC-1 to hMSC-5 in order of GR, and the hMSC-1 to hMSC-5 were from a 22-year-old black man, 20-year-old white man, 39-year-old black man, 29-year-old white woman, and 41-year-old white women, respectively. GR and colony-forming efficiency (CFE) for hMSC-1 to hMSC-5 and hFBs were 0.52 times/day and 9.6%, 0.35 times/day and 2.6%, 0.32 times/day and 2.0%, 0.30 times/day and 1.7%, 0.16 times/day and 0.3%, and 0.73 times/day and 0.0%, respectively. CFE showed a positive correlation with GR (*r* = 0.929, *P* = 0.022) (Figure 1(a)).

### 3.2. Proteins Derived from Culture Supernatant

To evaluate the protein secretion from MSCs relating to cartilage repair, antianabolic and catabolic factors for cartilage in culture supernatant were chosen for assessment. In the assessment of protein expression using ELISA, the anabolic factors TIMP-1, TIMP-2, TGF-*β*1, TGF-*β*2, PDGF-AA, HGF, and IGF-1 and the catabolic factors IL6, IL8, SDF-1a, MMP13, VEGF, MCP-1, MMP1, and MMP-3 were detected in the culture supernatant for each donor (Table 2). On the other hand, we were not able to validate protein expression for BMP-2, BMP-7, IL-4, IL-10, FGF-2, IFN*γ*, IL1*β*, IL2, IL17, MMP9, RANTES, TNF*α*, MIP1*α*, MIP1*β*, MIP3*α*, and TGF*α*. The 15 factors out of 31 could be detected using ELISA.

### 3.3. Percentage of Metachromasy in Pellets

**Figure 2:**
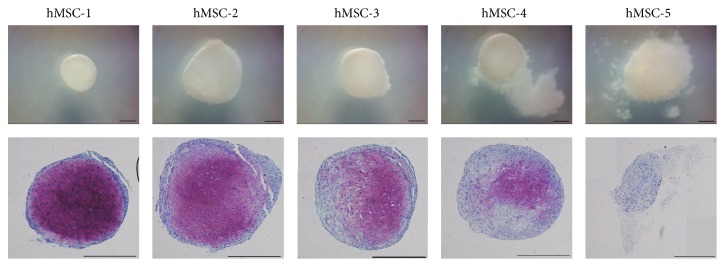
Percentages of metachromasy in pellets of hMSC-1 to hMSC-5 were 69.3%, 46.16%, 32.8%, 12.4%, and 0.0%, respectively. Scale bars, 500 *μ*m.

PMP of hMSC-1 to hMSC-5 were 69.3%, 46.16%, 32.8%, 12.4%, and 0.0%, respectively (Figure 2). The capacity for the production of extracellular matrix, demonstrating chondrogenic differentiation, was positively correlated with GR and CFE (GR: *r* = 0.951, *P* = 0.013; CFE: *r* = 0.878, *P* = 0.050) (Figures 1(b) and 1(c)).

### 3.4. Real-Time PCR Assays of Pellets

*COLII* gene expressions in hMSC-1 to hMSC-5 were 1.00, 1.08, 0.80, 0.63, and 0.00, respectively. Gene expression of *COL II* showed a correlative trend with CFE (*r* = 0.592, *P* = 0.293) and a correlation with GR (*r* = 0.840, *P* = 0.075) and PMP (*r* = 0.860, *P* = 0.062) (Figures 1(d), 1(e), and 1(f)). These findings indicate that chondrogenic capacity of hMSCs is positively correlated with proliferation capacity.

### 3.5. Histological Evaluation of Cartilage Repair

**Figure 3:**
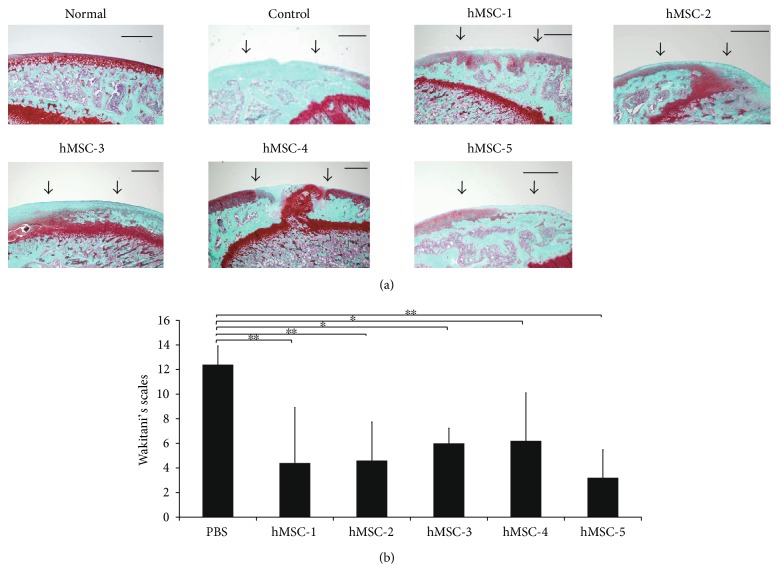
(a) Histological findings with Safranin-O/Fast green staining at 12 weeks after injection of hMSCs into the cartilage defect models. Scale bars, 500 *μ*m. (b) Assessment of the five different donors using Wakitani's scales (^∗∗^*P* < 0.01, ^∗^*P* < 0.05).

In the control group, the chondral defect area was not stained with Safranin-O. The mean histological score of the control samples was 12.40 ± 1.52 (SD) points. In hMSC-1, hMSC-2, and hMSC-5, the chondral defect area was partially stained with Safranin-O. However, in hMSC-3 and hMSC-4, the margins of the defect were irregular and the repair cartilage was composed of fibrous tissue. In hMSC-1 to hMSC-5 groups, the mean histological score was 4.4 ± 4.51 (SD), 4.6 ± 3.13 (SD), 6.0 ± 1.22 (SD), 6.2 ± 3.90 (SD), and 3.2 ± 2.28 (SD), respectively (Figure 3).

### 3.6. Gene Expression of hMSCs Assessed by 3D-Gene Human Oligo Chip 25k and TaqMan Low-Density Array

Of 24,460 mRNAs analyzed by 3D-Gene, we detected mRNA expressions of *MYBL1* (MYB proto-oncogene like 1) and *RCAN2* (regulator of calcineurin 2) related to proliferation capacity *in vitro* and *HLA-DRB1* (major histocompatibility complex, class II, DR beta 1) and *TMEM155* (transmembrane protein 155) related to cartilage repair *in vivo* (Table 3). Of 768 miRNAs analyzed by TLDA, we detected miR-486-3p, miR-148b, miR-93, and miR-320B in cartilage repair *in vivo* (Table 4). The gene expression in hMSCs from each donor was evaluated for fold changes compared with the gene expression in hFBs as a control for normalization. In the assessment of genes that were not detected in hFBs, the lowest gene expression in hMSCs was used as a control for normalization.

### 3.7. Relation In Vitro

The hMSCs from the five donors were divided into two groups of hMSC-1 to hMSC-3 and hMSC-4 to hMSC-5 according to their results in chondrogenic differentiation. In the toluidine blue staining and RT-PCR assays of pellets from hMSCs, hMSC-1 to hMSC-3 showed rich, whereas hMSC-4 and hMSC-5 showed poor, production of extracellular matrix. In addition, hMSC-1 to hMSC-3 showed good capacity, whereas those of hMSC-4 and hMSC-5 showed poor capacity, for cell proliferation. The mRNAs and miRNAs that could be divided into two groups of hMSC-1 to hMSC-3 and hMSC-4 to hMSC-5 according to their results in chondrogenic differentiation were selected and assessed for their correlation with chondrogenic differentiation.

In the assessment of mRNAs and miRNAs, the expression of *MYBL1* was higher and that of *RCAN2* lower in hMSCs from the three donors with good cell proliferation and production of extracellular matrix than in hMSCs from the other two donors. In addition, protein expression levels of both TIMP-1 and VEGF were negatively correlated with cell proliferation and production of extracellular matrix (Table 5).

### 3.8. Relation In Vivo

The hMSCs from the five donors were divided into two groups of hMSC-1, hMSC-2, and hMSC-5 and hMSC-3 to hMSC-4 according to their results in evaluation of cartilage regeneration using Wakitani's scales. The mRNAs and miRNAs that could be divided into two groups of hMSC-1, hMSC-2, and hMSC-5 and hMSC-3 to hMSC-4 according to their results in cartilage regeneration were selected and assessed for their correlation with cartilage regeneration.

The expressions of *MYBL1*, *RCAN2*, TIMP-1, and VEGF showing relevance with cell proliferation and chondrogenic differentiation had no correlation with cartilage repair *in vivo*. On the other hand, the expression of *HLA-DRB1* was higher and that of *TMEM155*, miR-486-3p, miR-148b, miR-93, and miR-320b lower in hMSCs from the three donors that showed good cartilage repair *in vivo* than in hMSCs from the other two donors (Table 6). However, protein expression levels of anabolic and catabolic factors for cartilage were not correlated with cartilage repair *in vivo*.

Finally, there was no correlation between the cell proliferation and production of extracellular matrix *in vitro* and the cartilage repair *in vivo*.

## 4. Discussion

This study demonstrated that the mRNA expression of *MYBL1* was positively correlated with proliferation and cartilage differentiation of hMSCs and that the mRNA expression of *RCAN2* and the protein expressions of TIMP-1 and VEGF were negatively correlated with proliferation and cartilage differentiation of MSCs. However, we also showed that MSCs from all five donors had the capacity to promote cartilage repair *in vivo* regardless of their capacity for proliferation and cartilage differentiation. The mRNA expression of *HLA-DRB1* was positively correlated with cartilage repair *in vivo*, and the mRNA expression of *TMEM155* and expressions of miR-486-3p, miR-148b, miR-93, and miR-320b were negatively correlated with cartilage repair.

*In vitro*, the capacity for chondrogenic differentiation, which is an index of extracellular matrix production, was high in cells with high proliferation, as indicated by CFE and the GR. However, we found that the cell proliferation and the chondrogenic differentiation cannot be used for the quality assessment of the MSCs based on the efficacy of cartilage repair *in vivo*. Thus, CFE, the gene expressions of *MYBL1* and *RCAN2*, and the protein expressions of TIMP-1 and VEGF can be used for the quality assessment of the MSCs based on the capacity of proliferation and chondrogenic regeneration, but not for the quality assessment of the MSCs based on the efficacy of cartilage repair. Previous studies reported that the tissue regeneration promoted by MSC transplantation might be mediated predominantly through the indirect paracrine mechanisms rather than the direct regeneration from transplanted MSCs [28–31]. Our previous study of intra-articular injection of green fluorescent protein (GFP) expressing rat MSCs into a rat cartilage defect model also showed that GFP-positive cell could be observed at the injured site at four weeks after the treatment but could not be detected in the posttreatment specimens at eight and 12 weeks [5]. This might be the reason for discrepancy between the *in vitro* chondrogenic capacity of MSCs and the cartilage repair *in vivo*. On the other hand, the expression levels of *HLA-DRB1*, *TMEM155*, miR-486-3p, miR-148b, miR-93, and miR-320b might be used for the quality assessment of MSCs based on the efficacy of cartilage repair. *HLA-DRB1* is part of a family of genes called the human leukocyte antigen (HLA) complex that has a critical role in the immune system. *HLA-DRB1* was reported to have participated in the pathopoiesis of rheumatoid arthritis [32, 33]. However, the function of *HLA-DRB1* in MSCs has not been reported. The function of TMEM in MSCs is also unknown. MiR-93 has been implicated in multiple cell processes, including proliferation, apoptosis, invasion, and extracellular matrix degradation [34–37]. Jing and Jiang reported that miR-93 is lower in human degenerative nucleus pulposus tissues and that its level is associated with disc degeneration grade. In addition, overexpression of miR-93 increases expression of type II collagen by targeting MMP3 and might thereby promote cartilage repair [38]. On the other hand, the functions of miR-486-3p, miR-148b, and miR-320b relating to MSCs or cartilage have not been previously studied. We found expression of specific mRNAs and miRNAs in hMSCs to be related to the capacity for cartilage regeneration. These genes might be used for the quality assessment of hMSCs before their use in treatment for cartilage repair.

In this study, the cartilage repair *in vivo* after MSC transplantation was incomplete. The xenograft of human MSCs to nude rats might be the reason of insufficient repair of articular cartilage. Another possible reason for insufficient repair of articular cartilage is the use of purchased hMSCs. The commercialized hMSCs were extremely expanded and frozen. This might have an undesirable influence on the quality of hMSCs for cartilage repair. In the next step, the qualities of hMSCs from the patients who take part in clinical trials should be assessed in the same way.

## 5. Conclusions

The cell proliferation and chondrogenic differentiation of hMSCs *in vitro* have no correlation with cartilage regeneration *in vivo*. On the other hand, we found expression of *HLA-DRB1*, *TMEM155*, miR-486-3p, miR-148b, miR-93, and miR-320B in hMSCs to be related to the capacity for cartilage regeneration. These factors might be useful for the quality assessment of hMSCs before their use in treatment for cartilage repair.

## Figures and Tables

**Table 1:** Primer sequences used for qPCR.

Gene	Primer sequence (5′ → 3′)
Type II collagen	GGCAATAGCAGGTTCACGTACA
	CGATAACAGTCTTGCCCCACTT
Type X collagen	CAAGGCACCATCTCCAGGAA
	AAAGGGTATTTGTGGCAGCATATT
Aggrecan	TACGAAGACGGCTTCCACCA
	CTCATCCTTGTCTCCATAGC
SOX9	GTACCCGCACTTGCACAAC
	GTAATCCGGGTGGTCCTTCT
CD44	AAGACACATTCCACCCCAGT
	GGTTGTGTTTGCTCCACCTT
GAPDH	ATGGGGAAGGTGAAGGTCG
	TAAAAGCAGCCCTGGTGACC

**Table 2:** Secretional capacity of proteins per 10,000 hMSCs (pg/10,000 hMSCs).

	TIMP-1	TIMP-2	TGF-*β*1	TGF-*β*2	PDGF-AA	HGF	IGF-1	
hMSC-1	5841.6	2790.4	192.3	19.8	1.1	2.5	17.7	
hMSC-2	12129.6	3714.5	283.6	31.8	2.0	4.3	44.3	
hMSC-3	14791.6	4227.6	333.2	40.5	2.1	1.4	64.8	
hMSC-4	23319.9	4394.7	367.0	38.5	1.7	85.8	44.4	
hMSC-5	41621.9	7640.3	479.5	54.6	4.2	60.3	80.8	
hFB	5879.2	3176.6	128.9	11.6	0.5	0.0	27.4	

	IL6	IL-8	SDF-1a	MMP13	VEGF	MCP-1	MMP-1	MMP-3

hMSC-1	123.9	0.4	150.1	1.7	449.8	30.0	55.6	10.4
hMSC-2	355.8	1.4	301.8	2.4	513.7	54.8	7.5	8.7
hMSC-3	606.8	1.1	278.8	3.1	742.0	59.0	32.1	22.8
hMSC-4	314.7	5.0	378.6	16.1	789.3	133.0	42.8	12.6
hMSC-5	966.5	8.4	284.5	4.1	1253.7	322.0	99.3	75.5
hFB	58.4	1.6	380.5	0.5	49.5	81.5	1096.2	1279.9

**Table 3:** Changes in mRNA expression in hMSCs.

	Gene	hMSC-1	hMSC-2	hMSC-3	hMSC-4	hMSC-5
In vitro	MYBL1	5.44	4.38	4.76	3.33	2.97
	RCAN2	0.07	0.07	0.08	0.14	0.15

In vivo	HLA-DRB1	0.07	0.15	0.28	0.45	0.03
	TMEM155	4.25	4.16	3.37	2.03	4.86

Fold changes.

**Table 4:** Changes in miRNA expression in hMSCs.

	Gene	hMSC-1	hMSC-2	hMSC-3	hMSC-4	hMSC-5
In vivo	miR-486-3p	2.12	1.55	0.88	1.00	3.11
	miR-148b	2.37	3.85	1.00	1.98	6.57
	miR-93	4.16	4.27	3.60	2.76	4.55
	miR-320B	2.34	3.46	1.99	0.89	4.53

Fold changes.

**Table 5:** Correlation analysis of specific factors *in vitro*.

mRNA		CFE	GR	PMP
MYBL1	*P*	0.098	0.042^∗^	0.014^∗^
	*r*	0.808	0.891	0.947
RCAN2	*P*	0.271	0.122	0.032^∗^
	*r*	−0.614	−0.777	−0.909
Protein				
TIMP1	*P*	0.164	0.026^∗^	0.025^∗^
	*r*	−0.727	−0.923	−0.923
VEGF	*P*	0.179	0.030^∗^	0.034^∗^
	*r*	−0.711	−0.914	−0.906

*P* = probability, *r* = correlation coefficient, ^∗^*P* < 0.05.

**Table 6:** Correlation analysis of specific factors *in vivo*.

mRNA and miRNA		CFE	GR	PMP	Wakitani
HLA-DRB1	*P*	0.605	0.918	0.666	0.024^∗^
	*r*	−0.315	−0.065	−0.265	0.726
TMEM155	*P*	0.779	0.937	0.763	0.031^∗^
	*r*	0.175	−0.050	0.187	−0.911

miR-486-3p	*P*	0.932	0.645	0.766	0.008^∗^
	*r*	0.054	−0.282	−0.185	−0.966
miR-148b	*P*	0.567	0.301	0.444	0.045^∗^
	*r*	−0.347	−0.584	0.453	−0.887
miR-93	*P*	0.799	0.950	0.735	0.037^∗^
	*r*	0.158	−0.039	0.209	−0.900
miR-320B	*P*	0.725	0.490	0.784	0.032^∗^
	*r*	−0.218	−0.413	−0.171	−0.910

*P* = probability, *r* = correlation coefficient, ^∗^*P* < 0.05.
